# Exploring People’s Perception of COVID-19 Risk: A Case Study of Greater Jakarta, Indonesia

**DOI:** 10.3390/ijerph20010336

**Published:** 2022-12-26

**Authors:** Dicky C. Pelupessy, Yasuhito Jibiki, Daisuke Sasaki

**Affiliations:** 1 Faculty of Psychology, Universitas Indonesia, Kampus UI Depok, Depok City 16424, West Java, Indonesia; 2 International Research Institute of Disaster Science, Tohoku University, 468-1 Aoba Aramaki, Aoba, Sendai 980-8572, Japan; 3 Center for Integrated Disaster Information Research, The University of Tokyo, 7-3-1 Hongo, Bunkyo-ku, Tokyo 113-0033, Japan

**Keywords:** perception, COVID-19, family, community, Jakarta, Indonesia

## Abstract

This study aims to understand people’s perceptions of COVID-19 risk in Greater Jakarta, Indonesia. In response to the COVID-19 pandemic, the Indonesian government enacted a health protocol campaign and highlighted the community as an important unit of protocol compliance. We hypothesized that people’s perception of the likelihood of being infected with COVID-19 is associated with health protocol compliance at the community level and their perception of community resilience. As the number of infected persons drastically increased, the “family cluster” also became a significant issue in the pandemic response, especially in Indonesia. In this study, we explored both community and family aspects that influence people’s perceptions. We conducted an online survey in March 2021 with 370 respondents residing in the Greater Jakarta area. The respondents were classified into four age groups (20s, 30s, 40s, and 50-and-over), with gender-balanced samples allocated to each group. We used a questionnaire to measure the perception of COVID-19 risk along with the Conjoint Community Resiliency Assessment Measure (CCRAM). Multiple regression analysis revealed that family factors have a much larger influence on the individual perception of the likelihood of contracting COVID-19 than community factors. The results suggest that the link between family-level efforts against COVID-19 and individual-level perceptions cannot be separated in response to the pandemic.

## 1. Introduction

In the wake of the COVID-19 pandemic, certain countries have opted to focus their responses on the community level [1]. In Indonesia, the national government enacted the Large-Scale Social Restriction Policy (*Pembatasan Sosial Berskala Besar*: PSBB) in April 2020, followed by the Enforcement of Restrictions on Community Activities (*Pemberlakuan Pembatasan Kegiatan Masyarakat*: PPKM) in January 2021 [2]. These policies highlight “communities” as important units for health protocol implementation in Indonesia. As the COVID-19 situation was updated, the government enacted the Emergency PPKM (PPKM Darurat), Micro-Based PPKM (known as PPKM Mikro), and PPKM levels. These new regulations do not substantially alter the importance of communities in health protocol compliance, but rely heavily on the capacity of communities in response to the pandemic. Such a social background reminds us of the need to consider the concept of “community resilience.” As communities consist of individuals, communities and individuals cannot be understood separately. We assume that individuals perceive COVID-19 through their communities and resiliency.

In addition to the importance of the community-level perspective, during the second wave of COVID-19 infection in Indonesia (May–September 2021), “self-isolation” in each household was a critical issue in health protocol implementation in Indonesia. The number of infected individuals increased drastically during this period. Each family faced great difficulty in managing health protocols in the household in the event that one of the family members tested positive because the hospitals and other medical facilities were fully occupied.

In the Indonesian context, community-level efforts and family-level responses may influence individual perceptions, which, however, need to be verified. Furthermore, recent research has reported that some types of individual demographics may be related to perceptions of COVID-19. Extant literature, such as a study from Peru and China [3] and another from Italy [4], noted that risk perception is the basis for examining the decision-making and behavior of each individual. Bavel et al. [5] indicated that it is important to better understand the risk perception of COVID-19 under pandemic conditions that nobody had experienced before.

Thus, using statistical analysis, we aimed to examine whether these three factors (demographic, family, and community) are relevant to people’s perceptions of COVID-19. As the COVID-19 situation has evolved, the issue of family cluster or household transmission has emerged in many countries (e.g., in Italy [6], India [7], the UK [8], Madagascar [9], Switzerland [10]). However, these studies indicate that the effects caused by family clusters and household transmission have not yet been well investigated, and further research is required. Moreover, due to the boundaries of academic disciplines, findings of community-related studies and those of family-related studies have not been integrated. In Greater Jakarta, people face both family/household issues and communities at the same time. As the demographic variables are the basis of individual characteristics and a large amount of research, it is reasonable to include them to examine family/household and community factors. Therefore, our aim to study these three factors is justified. For that reason, we targeted people who lived in Greater Jakarta, Indonesia, which has been considered the epicenter of COVID-19 in Indonesia since the pandemic started in 2020 [11].

## 2. Literature Review

During the COVID-19 pandemic, many researchers have examined how people perceive this phenomenon as unprecedented in their lifetime [3,12]. While some studies have focused on psychological factors at the individual level, others have considered the family and community perspectives. Although individual demographics also have an effect on people’s perceptions, generalized and consistent tendencies have not yet been observed. Furthermore, the findings were based on results from different countries using different methodologies. We note such limitations but review earlier research that investigated people’s perceptions to build our hypotheses based on the synthesized empirical findings.

### 2.1. Individual Risk Perception and COVID-19

Researchers have used a variety of words, such as worry, anxiety, fear, stress, attitude, threat, and perception. A simple definition, such as “Risk perceptions are interpretations of the world” [13] (p. 3), would be replicable and provide a basis for our study.

According to one literature review [3], many studies have demonstrated a link between risk perception and COVID-19 prevention [14,15,16]. However, Monge-Rodríguez et al. [3] and Yıldırım et al. [16] showed moderate effects of risk perception on protective behavior compared with the results of de Bruin and Bennett [14]. Rubaltelli et al. [17] reported a weak positive correlation between anxiety and risk perception. Existing literature has noted that risk perception is the basis for examining the decision-making and behavior of each individual [3,4,18,19,20,21,22]. Lohiniva et al. [19] and Shahin and Hussien [21] argued that understanding public risk perception is critical for risk perception. As Bavel et al. [5] indicated, it is important to better understand risk perception during the pandemic that nobody had experienced before.

### 2.2. The “Family Cluster” in Indonesia

To the best of our knowledge, family-level infections have not been studied extensively in some countries. However, in Indonesia, the so-called “family cluster” problem was considered an urgent issue during the pandemic. *The Jakarta Post*, one of the most trustworthy media outlets in Indonesia, reported that the Jakarta Special Province Governor stated that the family cluster was one of the major causes of the surge in COVID-19 infection after long holidays in October and November 2020 [23]. Supriyati et al. also found that COVID-19 transmission mostly occurs in families in Indonesia [24]. Furthermore, Nasrudin et al. noted that individual anxiety caused by COVID-19 influences family-level compliance with health protocols [25].

### 2.3. Role of the Community

Some studies have examined collectivistic (rather than individualistic) aspects of COVID-19 responses. There is a theoretical assumption that risk is perceived through the lens of group membership [26]. According to Stevenson et al. [27], the “group process” [28,29] and “group-level nature” [30,31] are crucial perspectives for understanding the current pandemic situation, and community identity plays a pivotal role in effective behavioral responses to COVID-19 [27]. Moreover, one study [32] found that social support eased the negative mental health impact of COVID-19. These findings suggest that local settings, including the communities to which people belong, are important for examining how people perceive COVID-19.

### 2.4. Individual Risk Perception and Demographic Factors

#### 2.4.1. Age

Some studies indicate that age affects the relationship between individual risk perceptions and age. Chan et al. demonstrated that the elderly are less likely to worry about COVID-19 [33]. Similarly, Megatsari et al. found that older individuals experienced less anxiety [34]. Savadori and Lauriola found that younger participants were less worried about getting infected with the coronavirus [4], whereas Adiyoso and Wilopo found that younger individuals showed a stronger relationship with risk perception [35]. Harapan et al. demonstrated that age was a significant predictor of the risk of infection and the study participants aged between 21 and 30 years had the highest perceived risk [36]. Bernabe-Valero et al. showed that younger people exhibit higher stress levels [12]. Based on our review, it seems difficult to determine whether older or younger individuals show a consistent tendency, even though age itself matters. However, other studies have not clearly indicated that age is relevant to individual risk perception [3,37,38].

#### 2.4.2. Gender

Only a few studies indicate that gender is unrelated to risk perception. Two studies analyzed the perception of the risk of contracting coronavirus, but their analysis suggested that gender was not a significant predictor of risk perception [36,37]. In contrast, most studies have reported that women show more negative responses to risk perception [3,4,12,15,16,34,39,40,41,42,43].

#### 2.4.3. Education

Three studies [12,34,37] demonstrated a similar tendency in the effect of educational level on risk perception, such that those with lower educational levels showed higher levels of negative feelings about COVID-19. However, two studies indicated that education was not significant for risk perception [4,36].

#### 2.4.4. Marital Status

Harapan et al. revealed that the perceived risk of infection was lower among married than unmarried respondents [36]. Similarly, Bernabe-Valero et al. showed that individuals who were single exhibited higher stress levels [12].

## 3. Hypotheses

**Figure 1:**
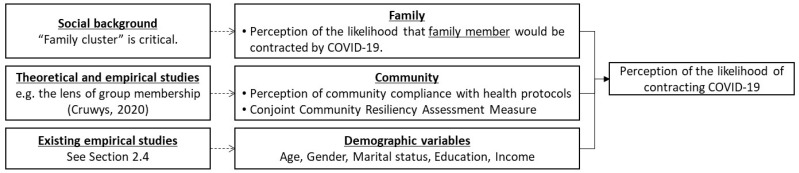
Hypothetical framework of our analysis.

Based on the empirical findings summarized in the literature reviewed, demographic, family, and community-level factors are relevant to people’s perceptions of COVID-19. In this study, we define people’s perception of the likelihood of contracting COVID-19 at the individual level as the dependent variable (see Table 1 and Figure 1).

The first independent variable was the family’s role. We used the variable of perception of the likelihood that a family member would contract COVID-19. We assume that people perceive their own likelihood of contracting COVID-19 to be greater when they perceive that of their family members to be greater.

The second independent variable was the community. We used two types of variables: perception of community compliance with health protocols and joint community resilience assessment measure (CCRAM). While the former directly reflects how people perceive a health response, the latter assesses more generalized crisis contexts. The details of the CCRAM are explained in the next section.

We set some types of demographics as the first independent variables. As noted earlier, it is difficult to determine whether older or younger individuals show a consistent tendency, even though age itself matters. Female respondents reported a more negative response to perceived risk. As the effects of education and marital status have not yet been determined, we sought to verify whether they were significant.

We used multiple regression analysis to control for the effects of demographic variables.

To clarify our terminology, we define communities as *kelurahan*s (towns) that could be treated as a unit of locality that is smaller than the city but larger than the neighborhood. Hence, in some ways, it can be considered a town.

## 4. Methods

Although the survey implementation, data, and CCRAM measurements were the same as those of Pelupessy et al. [44], the research purpose in this article is original, and the hypothetical idea has no duplication with Pelupessy et al. [44].

### 4.1. COVID-19 in Greater Jakarta

Greater Jakarta is one of the largest urban areas in the world [45]. As the COVID-19 pandemic continues, Greater Jakarta has faced an increasing number of infections [11].

Communities are among the key actors in Greater Jakarta’s response to the pandemic. Pangaribuan and Munandar argued that governmental organizations in Greater Jakarta faced significant difficulty in responding to the community’s non-compliance with health protocols under PSBB implementation [46]. Yakhamid and Zaqi indicated that the Jakarta Special Province government achieved PPKM Darurat due to the synergy between community compliance in implementing health protocols and the achievements of the vaccination program [47].

Family clustering in Greater Jakarta is also a critical issue. Handayani et al. found that the stricter health protocol in Greater Jakarta was rarely applied, especially by those who lived in the same house, resulting in family members infected with the rest of their family from workplace cluster cases [48].

Both community and family aspects are observed in Greater Jakarta; therefore, we think that it is reasonable to test our hypothesis using Greater Jakarta as a case study. 

### 4.2. On-Line Survey Implementation and Our Respondents

We conducted an online survey in March 2021 with 370 respondents residing in the Greater Jakarta area. The period in which we implemented the online survey was immediately after the Indonesian people experienced the first wave of COVID-19 (around February 2021). As the survey was conducted during the period between the first wave of COVID-19 in Indonesia and the second wave (May–September 2021), the social situation around Greater Jakarta was relatively calm, and the respondents were able to participate in the survey.

Participants (*n* = 370) were adults aged 18–59 years (*M* = 37.7, *SD* = 10.5 years). They were recruited using quota sampling while maintaining an equal proportion of male and female participants to account for gendered perceptions. Respondents were classified into four age groups (20s, 30s, 40s, and 50-and-over). In the 50-and-over age group, 35 respondents were allocated to the male and female groups. All participants completed online and web-based questionnaires.

To the best of our knowledge, the surveys of the existing literature were largely conducted online, except for [33,37]. Considering the current pandemic situation, online surveys are considered the most feasible. It should be noted that the samples could be biased, and such a methodological challenge should be a major concern in future research.

To identify the sample size, we adopted the following steps: Based on the fact that the population size of Greater Jakarta is sufficiently large, we considered the required sample size in the interval estimation of the population proportion. We set the margin of error to 5%, the confidence level to 95%, and the population proportion to 0.5. We identified 385 samples as target values. Considering that the older generation seems less familiar with using online survey platforms, we assumed that fewer survey participants were in the older generation than the younger generation. Practically, we secured 70 samples in the age bracket of over 50s, while we received 100 samples in their 20s, 30s, and 40s. Finally, we collected 370 samples in total using quota sampling.

A summary of the demographic information is presented in Table 2. Regarding the primary occupation of the respondents, 21.6% were in the retail sector, the largest sector in the sample. Housewives and those working in the manufacturing sector accounted for 10.8% of the respondents. Others were employed in construction, transportation, education, government, and other sectors.

### 4.3. Measures

To analyze perceptions of COVID-19, we administered the CCRAM. As reviewed in Section 2.4, some studies suggest the importance of collectivistic (rather than individualistic) thinking in the case of COVID-19. Although there are several instruments to assess how people perceive matters through a collectivistic lens, we used the CCRAM in this study. The COVID-19 pandemic, caused by community transmission, highlights the significance of the community. The CCRAM is not only a community resilience measurement tool but also one fit for use in both daily and emergency/crisis situations in terms of community-related parameters [49]. Several recent studies have measured community resilience related to and during the COVID-19 pandemic using the CCRAM [50,51,52].

The CCRAM is a measure of community resilience. Leykin et al. [53] developed it to establish an integrated multidimensional instrument to assess community resilience and later [54] tested whether their measurement was robust as a psychological indicator showing peoples’ evaluations of their communities’ capacity to respond to emergencies in diverse contexts. It assesses the strength of five important dimensions of community function (leadership, collective efficacy, preparedness, place attachment, and social trust) that can be used to profile and predict community resilience [53,54,55].

The CCRAM assesses leadership factors in community resilience through six items representing general faith in decision-makers, specific faith in local leaders, perception of fairness in the way local authority provides services, and functioning of the community [53]. Collective efficacy was evaluated using five items representing collective efficacy, support, involvement in the community, and mutual assistance [53]. The collective efficacy items echo arguments that they comprise a composite of mutual trust and a shared willingness to work for the common good of a neighborhood [56,57]. The preparedness factor comprises four items representing family and community acquaintances with emergency situations and a view of the town’s preparedness for emergency situations [53]. The place attachment factor is composed of four items representing emotional attachment to the community, sense of belonging, pride in the community, and ideological identification with the community [53]. This perspective regarding place attachment aligns well with the assertions of Manzo and Perkins, who, based on a cross-disciplinary literature review, pointed out that individuals’ feelings toward their place are connected to community-level perceptions [58]. Finally, the social trust factor is composed of two items representing trust and the quality of relationships between members in the community [53].

In this study, we used a 21-item self-report CCRAM questionnaire. Each item was measured on a five-point Likert scale, and the answers provided scores. The total CCRAM score was calculated as the sum of all the scores for each item. These five dimensions were generated in accordance with Leykin et al.’s categorization [53]. For example, “leadership” consists of six items, and its value is calculated as the sum of the scores of these six items. Details of the 21 items of the CCRAM, the 5 dimensions, and their basic statistics are presented in Appendix A and Appendix B.

### 4.4. Models

Multiple regression analysis was conducted to verify our hypotheses. The five ways (or written “models” hereinafter) of the multiple regression analysis are presented in Table 3. In the multiple regression analysis, the dependent variable “Perception of the likelihood of contracting COVID-19” was consistently used in all analyses.

The independent variables “Demographic” (age, gender, marital status, education, and household income), “Family” (perception of the likelihood that family member would contract COVID-19), and “Community” (perception of community compliance with health protocols) were used in all analyses.

Only CCRAM was exceptional: In Models 1 and 2, we used the total CCRAM score. Models 3 and 4 tested the five dimensions of the CCRAM (leadership, collective efficacy, preparedness, place attachment, and social trust). In Model 5, we input all 21 CCRAM items.

For multiple regression analysis, the forced imputation method was adopted in Models 1 and 3. A stepwise method was used for Models 2, 4, and 5.

While our analysis provides baseline data based on a survey carried out in March 2021, continuing research is necessary to understand people’s perceptions more deeply, considering the evolving COVID-19 situation.

## 5. Results

Multiple regression analysis was conducted to verify whether the three hypothetical factors (demographic, family, and community levels) were relevant to people’s perceptions of COVID-19 (see Table 3). 

Among the five models, Model 5 was identified as the best fitting- model with reference to its highest value of the adjusted *R*^2^, which was 0.706. As the value of the adjusted *R*^2^ in all models was approximately 0.7, our models reasonably explained the dependent variable. Regarding the effects of demographic variables, only “marital status” was significant for the dependent variable, such that married individuals perceived themselves as more likely to contract COVID-19. This result is inconsistent with earlier findings [12,36]. However, “marital status” was significant in Models 1 and 3, but not in Model 5, the best-fitting model. Although some researchers have reported that age, sex, education, and income may be relevant to risk perception, our analysis did not reveal any significant associations.

The significance of the family level aspect was prominent in multiple regression analysis. In all models, the value of the standardized coefficient (β) for the perception of the likelihood that family members would contract COVID-19 was the highest. These results indicate that those who think that their family members will be infected with COVID-19 perceive that they are also likely to be infected, corroborating Nasrudin et al. [25]. As indicated in the previous paragraph, “marital status” was significant. This result indicates that married individuals are more likely to contract COVID-19, reflecting that the family aspect is an important factor.

For the community-level factor, the independent variable “perception of health protocol compliance at the community level” did not demonstrate significance in any model. By contrast, the collective efficacy dimension of the CCRAM and an item of the social trust dimension were significant. In Models 3, 4, and 5, the collective efficacy dimension was significant. However, the value of the standardized coefficient (β) was positive, indicating that those who maintained higher collective efficacy tended to think that they were more likely to contract COVID-19. However, these results are contradictory. In our interpretation, due to the seriousness of the pandemic, even though someone maintains collective efficacy, such a degree of perception cannot overcome its influence. The social trust dimension of CCRAM was significant only in Model 5, indicating that those who put greater trust in various groups perceived that they were less likely to be infected by COVID-19. This result implies that higher social trust can reduce negative perceptions of COVID-19.

## 6. Discussion and Conclusions

This study aimed to examine people’s perceptions of COVID-19. Based on our multiple regression analysis, the “family” factor seems highly relevant to people’s perceptions. The community-level factor showed significance, but compared with the family factor, it did not contribute much to people’s perceptions. However, as noted earlier, the findings should be verified using case studies with different data for greater generalizability.

In future research, we will highlight the definition of communities and characteristics of the data. First, in the present study, we defined “communities” as “*kelurahan*” (towns). However, if we adopt a different definition of communities, such as neighborhood associations (locally known as *Rukun Tetangga* and *Rukun Warga*) or traditional units *kampung*, we might obtain different results. Second, data characteristics should be carefully considered. The period of survey implementation may have affected the data and results. If we carried out another survey in a relatively calm situation or if the capacities of communities were improved gradually, the family factor would not be much stronger. In terms of survey implementation, our data were collected through an online survey; however, this method has limitations. As older people may be less familiar with the internet, they may hesitate to participate online. Even though we controlled for age in the multiple regression analysis, both the original data and the potential respondents for online surveys were biased to some extent. Future research needs to consider the possibility of different sampling techniques and explore whether qualitative data analysis can be a useful method to supplement the limitations of quantitative methods.

Based on our analysis, we adduce two practical implications: the identification of “communities” and “family” affairs. For more effective community-level interventions, it is important to consider which types of “communities” should be targeted. As described above, town and neighborhood associations can be distinguished. In the best-fitting model of our multiple regression analysis, the independent variable “The relations between the various groups in my town are good” was significant. This result implies that there are subgroups within a town, and, thus, it would appear meaningful to specifically identify how these subgroups contribute to the response to COVID-19. We hardly found that infected families were isolated without assistance, even though they faced difficulties. Communities and their subgroups may have filled this gap to achieve seamless assistance. Kuno [59], based on his observation in the central area of Jakarta, reported a “neighborhood lockdown” guided by neighborhood associations. Hosobuchi [60], also based on her study in Jakarta during the COVID-19 pandemic, introduced activities at neighborhood associations for juvenile delinquency prevention and provided religious education programs. This empirical evidence suggests that communities and their subgroups may have proactively contributed to the management of local needs, and more detailed studies are necessary in future research.

The results of our multiple regression analysis indicate that “I (person A) would be more likely to contract COVID-19 if my family member (person B) contracted COVID-19”. Person B’s perception of the likelihood of contracting COVID-19 was highly dependent on the health condition of person A as B’s family member. This interpretation suggests a chain reaction in each family and highlights the importance of mutuality among the family members.

As broader implications of the findings for similar contexts of other pandemics, not just the COVID-19 pandemic, we consider two aspects: the necessity of long-term studies and more standardized surveys. First, the necessity of longer-term studies reflects the longer and evolving process of COVID-19 expansion. Approximately three years have passed since the COVID-19 pandemic began; we have been experiencing some changes in its expansion stages. In the early stage, social lockdown and public health protocol compliance at the community level were our main concerns. Then, vaccination became a critical issue. However, not many people were better aware of family cluster/household transmission in the beginning. Such dynamics of the current pandemic, noting that the virus mutation continues, may have complicated effects on people’s perceptions. Long-term studies are necessary to better understand the evolving nature of the disease. Second, more standardized surveys should be conducted in the future. Facing unprecedented social deficiencies, many surveys, including ours, have been conducted worldwide using different methodologies without common definitions of critical terminologies. Although each study has the right to determine its own methods and concepts, standardized surveys aiming at more harmonized efforts to monitor people’s perceptions should be a challenge in future research.

## Figures and Tables

**Table 1:** Types of the dependent and independent variables.

Variable Name	Types of Variables
**Dependent variable**	
Perception of the likelihood of contracting COVID-19 at the individual level	Five-point Likert scale, 1 = very unlikely, 5 = very likely
**Independent variable**	
Perception of the likelihood that a family member would contract COVID-19	Five-point Likert scale, 1 = very unlikely, 5 = very likely
Perception of community compliance with health protocols	Five-point Likert scale, 1 = very unlikely, 5 = very likely
CCRAM *^1^	Continuous variable
Age	Continuous variable
Gender	Binary, 1 = male, 0 = female
Marital status	Binary, 1 = married, 0 = not married
Education *^2^	Quantitative variable, 1 = the lowest (Elementary), 6 = the highest (Post-graduate)
Household income	Quantitative variable, 1 = the lowest (< IDR 1,500,000), 7 = the highest (>IDR 10,000,000)

*^1^ CCRAM: Conjoint Community Resiliency Assessment Measure. *^2^ We interpreted the education variable as the degree of educational level (relatively higher to lower), and the level was dealt with as a quantitative variable. In Table 2, the elementary and the junior high school were merged due to very small-sized samples. Five (1.4%) and eleven (3.0%) respondents had elementary and junior high education, respectively.

**Table 2:** Demographic statistics summary (*n* = 370).

Variable	Percent	Variable	Percent
**Gender**		**Marital status**	
Male (*n* = 185)	50.0	Married (*n* = 239)	64.6
Female (*n* = 185)	50.0	Unmarried (*n* = 131)	35.4
**Age**			
20–29 (*n* = 100)	27.0		
30–39 (*n* = 100)	27.0	**Household Income**	
40–49 (*n* = 100)	27.0	<IDR 1,500,000 (*n* = 20)	5.4
>50 (*n* = 70)	18.9	IDR 1,500,000–2,499,999 (*n* = 21)	5.7
**Education**		IDR 2,500,000–3,499,999 (*n* = 28)	7.6
Below High School level (*n* = 16)	4.4	IDR 3,500,000–4,999,999 (*n* = 42)	11.4
Senior High (*n* = 101)	27.3	IDR 5,000,000–7,499,999 (*n* = 74)	20.0
Diploma 1–4 (*n* = 52)	14.1	IDR 7,500,000–9,999,999 (*n* = 60)	16.2
University (Bachelor’s) (*n* = 176)	47.6	>IDR 10,000,000 (*n* = 125)	33.8
Post-graduate (Master’s and Ph.D.) (*n* = 25)	6.7		

**Table 3:** Results of the multiple regression analyses. Note: Dependent variable for all models was “perception of the likelihood of contracting COVID-19”.

Model 1. Forced imputation method				
	Standardized Coefficient (β)	*t*-value	*p*-value	VIF
Constant		0.846	0.398	
Age	−0.051	−1.705	0.089	1.117
Gender	0.026	0.913	0.362	1.019
Marital Status	0.069	2.203	0.028	1.205
Education	0.050	1.528	0.127	1.310
Household Income	−0.036	−1.049	0.295	1.483
Family would be infected	0.838	28.404	0.000	1.068
Perception of health protocol compliance at the community level.	−0.029	−0.933	0.351	1.170
CCRAM Total Score	0.038	1.233	0.219	1.194
*R*	0.840			
adj *R*^2^	0.699			
*n*	370			
Model 2. Stepwise method				
	Standardized Coefficient (β)	*t*-value	*p*-value	VIF
Constant		4.307	0.000	
Family would be infected.	0.835	29.069	0.000	1.000
*R*	0.835			
adj *R*^2^	0.696			
*n*	370			
Model 3. Forced imputation method				
	Standardized Coefficient (β)	*t*-value	*p*-value	VIF
Constant		0.764	0.446	
Age	−0.054	−1.808	0.072	1.121
Gender	0.019	0.642	0.521	1.035
Marital Status	0.068	2.176	0.030	1.208
Education	0.044	1.329	0.185	1.335
Household Income	−0.029	−0.831	0.406	1.495
Family would be infected.	0.838	28.491	0.000	1.075
Perception of health protocol compliance at the community level	−0.024	−0.791	0.429	1.191
CCRAM_1. Leadership	−0.103	−1.729	0.085	4.426
CCRAM_2. Collective Efficacy	0.125	2.043	0.042	4.687
CCRAM_3. Preparedness	0.055	1.001	0.318	3.795
CCRAM_4. Place Attachment	0.026	0.615	0.539	2.236
CCRAM_5. Social Trust	−0.066	−1.390	0.165	2.801
*R*	0.844			
adj *R*^2^	0.703			
*n*	370			
Model 4. Stepwise method				
	Standardized Coefficient (β)	*t*-value	*p*-value	VIF
Constant		0.524	0.601	
Family would be infected.	0.839	29.248	0.000	1.006
Collective Efficacy	0.057	1.972	0.049	1.006
*R*	0.837			
adj *R*^2^	0.698			
*n*	370			
Model 5. Stepwise method				
	Standardized Coefficient (β)	*t*-value	*p*-value	VIF
Constant		1.849	0.065	
Family would be infected.	0.830	29.353	0.000	1.005
I can depend on people in my town to come to my assistance in a crisis. [Collective Efficacy]	0.119	3.867	0.000	1.195
The relations between the various groups in my town are good. [Social Trust] [Social Trust]	−0.065	−2.116	0.035	1.199
*R*	0.842			
adj *R*^2^	0.706			
*n*	370			

## Data Availability

The data presented in this study are available on request from the corresponding author.

## Appendix A. Basic Statistics of the Five Dimensions of CCRAM (*n* = 370)

	**Min**	**Max**	**Mean**	***SD***
CCRAM Total Sum	23	105	74.797	12.511
CCRAM: Leadership	6	30	21.51	4.17
CCRAM: Collective Efficacy	5	25	18.18	3.09
CCRAM: Preparedness	4	20	13.96	2.72
CCRAM: Place Attachment	4	20	13.61	2.64
CCRAM: Social Trust	2	10	7.54	1.39

## Appendix B. Basic Statistics of the 21 Items of CCRAM (*n* = 370)

	**Mean**	***SD***
1. The municipal authority (regional council) of my town functions well [1: Leadership]	3.66	0.83
2. There is mutual assistance and concern for others in my town [2: Collective Efficacy]	3.85	0.74
3. My town is organized for emergency situations [3: Preparedness]	3.40	0.85
4. I am proud to tell others where I live [4: Place Attachment]	3.61	0.87
5. The relations between the various groups in my town are good [5: Social Trust]	3.78	0.81
6. I have faith in the decision-makers in the municipal authority (regional council) [1: Leadership]	3.62	0.83
7. I can depend on people in my town to come to my assistance in a crisis [2: Collective Efficacy]	3.32	0.89
8. The residents of my town are acquainted with their role in an emergency situation [3: Preparedness]	3.67	0.78
9. I feel a sense of belonging to my town [4: Place Attachment]	3.61	0.79
10. There is trust among the residents of my town [5: Social Trust]	3.76	0.73
11. In my town, appropriate attention is given to the needs of children [1: Leadership]	3.50	0.87
12. There are people in my town who can assist in coping with an emergency [2: Collective Efficacy]	3.63	0.82
13. In my town, there are sufficient public protection facilities (such as shelters) [3: Preparedness]	3.34	0.89
14. I remain in this town for ideological reasons [4: Place Attachment]	3.11	0.92
15. I have faith in the ability of the elected/nominated head of my town to lead the transit from routine to emergency management of the town [1: Leadership]	3.58	0.82
16. I believe in the ability of my community to overcome an emergency situation [2: Collective Efficacy]	3.72	0.74
17. My family and I are acquainted with the emergency system of my town (to be activated in times of emergency) [3: Preparedness]	3.55	0.84
18. I would be sorry to leave the town where I live [4: Place Attachment]	3.29	0.91
19. The municipal authority (regional council) provides its services in fairness [1: Leadership]	3.52	0.90
20. The residents of my town are greatly involved in what is happening in the community [2: Collective Efficacy]	3.65	0.80
21. The residents of my town will continue to receive municipal services during an emergency situation [1: Leadership]	3.62	0.78
Note: “[]” at the end of each item refers to the names of the five dimensions. For example, “leadership” consists of six items, one of which is Item 1.		
